# Preclinical Evaluation of Electronic Health Records (EHRs) to Predict Poor Control of Chronic Respiratory Diseases in Primary Care: A Novel Approach to Focus Our Efforts

**DOI:** 10.3390/jcm13185609

**Published:** 2024-09-21

**Authors:** Fernando M. Navarro Ros, José David Maya Viejo

**Affiliations:** 1 Centro de Salud Malilla, Carrer de Malilla 52D, Quatre Carreres, 46026 Valencia, Spain; fnavarroyros@hotmail.com; 2 Centro de Salud de Camas, Santa Maria de Gracia 54, 41900 Camas, Spain

**Keywords:** asthma, COPD, electronic health records, clinical predictors, primary care

## Abstract

**Background/Objectives:** Managing chronic respiratory diseases such as asthma and chronic obstructive pulmonary disease (COPD) within the Spanish *Sistema Nacional de Salud* (SNS) presents significant challenges, particularly due to their high prevalence and poor disease control rates—approximately 45.1% for asthma and 63.2% for COPD. This study aims to develop a novel predictive model using electronic health records (EHRs) to estimate the likelihood of poor disease control in these patients, thereby enabling more efficient management in primary care settings. **Methods:** The Seleida project employed a bioinformatics approach to identify significant clinical variables from EHR data in primary care centers in Seville and Valencia. Statistically significant variables were incorporated into a logistic regression model to predict poor disease control in patients with asthma and COPD patients. Key variables included the number of short-acting β-agonist (SABA) and short-acting muscarinic antagonist (SAMA) canisters, prednisone courses, and antibiotic courses over the past year. **Results:** The developed model demonstrated high accuracy, sensitivity, and specificity in predicting poorly controlled disease in both asthma and COPD patients. These findings suggest that the model could serve as a valuable tool for the early identification of at-risk patients, allowing healthcare providers to prioritize and optimize resource allocation in primary care settings. **Conclusions:** Integrating this predictive model into primary care practice could enhance the proactive management of asthma and COPD, potentially improving patient outcomes and reducing the burden on healthcare systems. Further validation in diverse clinical settings is warranted to confirm the model’s efficacy and generalizability.

## 1. Introduction

In recent decades, factors such as globalization, urbanization, aging populations, and shifts in health policies have contributed to the rising prevalence of chronic diseases, now the leading cause of death worldwide [1,2]. Among these, chronic respiratory diseases (CRDs) such as asthma and chronic obstructive pulmonary disease (COPD) are particularly concerning due to their high mortality and morbidity rates [1,3]. These conditions not only significantly reduce patients’ quality of life but also place a substantial economic burden on healthcare systems, with global costs projected to reach USD 47 trillion by 2030 [1,4,5]. This economic impact reflects both direct healthcare costs and a 20% increase in Disability-Adjusted Life Years (DALYs) since 1990 [6].

Asthma and COPD are the most prevalent CRDs, affecting approximately 262 million and 212 million people globally, respectively [7]. The severity of these diseases is reflected in alarming statistics: over 1000 people die from asthma daily, and COPD-related deaths have increased by 26% since 1990, now accounting for 3.3 million deaths annually, making COPD the fourth leading cause of death worldwide [8,9,10,11]. Managing the complex needs of patients with CRDs, particularly those with asthma, COPD, and overlapping features of asthma and COPD (asthma–COPD overlap syndrome or ACOS) poses a significant challenge in medical practice. Inadequate diagnosis and misdiagnosis of asthma and COPD are common in primary care settings, often leading to suboptimal treatment and poor disease control [12,13,14,15].

Given these challenges, primary care plays a crucial role in managing chronic conditions. Effective primary care can enhance prevention strategies, improve treatment outcomes, and reduce the strain on secondary healthcare services [6,14,16]. Strong primary care systems can meet the clinical needs of most patients efficiently, whereas weak or inadequate systems are associated with higher risks of hospitalization and complications [2,17]. However, a significant proportion of asthma and COPD patients still do not achieve effective disease control. Approximately 45.1% of asthma patients and 63.2% of COPD patients have uncontrolled disease, highlighting the need for more comprehensive evaluation and management strategies [18,19,20,21,22].

Recent advancements in Spain, such as the implementation of electronic health records (EHRs) in the *Sistema Nacional de Salud* (SNS), offer a promising tool to improve healthcare delivery by reducing unnecessary procedures and ensuring timely access to updated clinical information across any regional health service [23,24]. EHRs provide a valuable opportunity to develop preclinical tools capable of screening patients at risk of poorer health outcomes more effectively. Such tools would allow healthcare professionals to prioritize patients with more severe prognoses and optimize the allocation of medical resources.

Therefore, in this study, we propose the use of predictive clinical variables derived from EHR data to assess the control of chronic respiratory diseases, such as asthma and COPD. This novel approach aims to perform effective preclinical screening of these patients, potentially enhancing disease management in primary care without increasing the demand for care.

## 2. Materials and Methods

### 2.1. Study Overview

Seleida is a multicenter, observational, non-interventional study designed to identify clinical variables that could predict poor control of asthma and COPD using EHR data. Data were randomly selected from digital clinical records of primary care centers in Seville and Valencia. The project was approved by the Ethics Committee of Valencia (CEIm 132.22, approval date 6 March 2023) and was registered in the *Portal de Ética de la Investigación Biomédica de Andalucía* through *the Sistema de Información de los Comités de Ética de la Investigación* (1140-N-23, approval date 12 September 2023), with the endorsement of the SEMERGEN Research Department (2023-00035, approval date 6 June 2023).

### 2.2. Data Source and Patient Selection

Patient data were anonymously extracted from the digital clinical history of the Spanish SNS between 1 May 2024 and 31 July 2024. Each patient was assigned a unique code to ensure confidentiality and protection of their personal data.

#### 2.2.1. Inclusion Criteria

The study included patients aged between 18 and 80 years with a confirmed clinical and/or spirometric diagnosis of asthma and those aged 40 to 80 years with a diagnosis of COPD, all registered in the SNS as of 31 December 2023. Furthermore, patients without a formal diagnosis recorded in the EHR, but who had received treatment for asthma or COPD for a minimum of three months per year over the preceding two years, were also considered eligible for inclusion.

#### 2.2.2. Exclusion Criteria

For the asthma cohort, patients classified at step 6 according to the treatment scale outlined in version 5.4 of the Guía Española para el Manejo del Asma (GEMA) [25] were excluded. Exclusion criteria applicable to both asthma and COPD cohorts included patients with a diagnosis of active cancer or those receiving palliative care, those undergoing biologic therapy, pregnant patients, and individuals with concurrent diagnoses of asthma and COPD or ACOS. Additionally, patients with conditions such as rheumatic diseases or other pathologies requiring regular systemic corticosteroid use, bedridden patients, and those participating in clinical trials were also excluded. These criteria were implemented to minimize the potential of confounding factors that could affect the control of respiratory diseases or the response to treatment, thereby ensuring a more homogeneous study population. To avoid selection bias, patients were selected from groups not assigned to the authors.

#### 2.2.3. Variables Collected

Clinical variables were extracted from EHRs to identify effective predictors of poorly controlled asthma or COPD. These variables included relevant demographic and anthropometric data (age, gender, height, weight and body mass index [BMI]), comorbidities and treatable traits that may affect respiratory disease control (smoking habit, obesity; sleep apnea; rhinitis or chronic rhinosinusitis, nasosinusal polyposis; drug, environmental and food allergies; bronchiectasis; gastroesophageal reflux; ischemic heart disease; heart failure; arrhythmias; hypercholesterolemia; hypertriglyceridemia; hypertension, patients undergoing non-cardiovascular β2 blocker or angiotensin-converting enzyme [ACE] inhibitor treatment; diabetes mellitus; patients undergoing glucagon-like peptide-1 [GLP1] agonist or sodium-glucose cotransporter-2 [SGLT2] inhibitor treatment; anxious–depressive disorder; anemia; osteoporosis; chronic kidney disease or peripheral arterial disease), and disease history (current treatment, number of daily inhalations; measurement of rescues with short-acting β-agonists [SABAs], short-acting muscarinic antagonists [SAMAs; Ipratropium Bromide]; days without treatment due to lack of prescription or dispensing; exacerbations in the last year; visits to the emergency department or physician’s office in the last year due to asthma or COPD; oral or parenteral corticosteroid therapy expressed as number of regimens and as equivalent doses of prednisone in the last year; antibiotics used for treating bronchitis or exacerbations expressed as number of courses and as number of days of treatment in the last year; eosinophilia). For both asthma and COPD, two additional variables were recorded: the province of origin and an identification number, which ensured patient anonymity. Additionally, asthma patients’ data were collected for the following variables: assessment of asthma severity (treatment step according to GEMA 5.4 [25]) and current treatment with inhaled corticosteroid (IC)–formoterol combinations (treatment, number of canisters and doses of each canister, and number of daily inhalations). In the case of COPD, data were collected on the ABE classification defined by the 2024 version of the Global Initiative for Chronic Obstructive Lung Disease (GOLD) [26] and the number of severe exacerbations (with hospital admission) in the last year.

The control variable was calculated for all patients based on a selection of parameters collected in the current evidence.

### 2.3. Model and Calculating Predictions

The current clinical characterization of asthma control is well defined, being based on three key areas: current symptoms, recent lung function, and exacerbations in the past year [25]. In contrast, the 2021 update of the Spanish COPD guideline (GesEPOC) proposes a control tool that has not yet achieved a broad international consensus [27]. Both approaches (asthma and COPD) have significant limitations in preclinical primary care studies due to difficulties in obtaining consistent lung function data and symptom evaluation using validated scales.

This pilot study aims to overcome these limitations by identifying variables within EHR associated with asthma and COPD control and developing a predictive model to estimate the preclinical probability of poor control. This model would allow a focus on patients at higher risk, facilitating proactive management and optimization of primary care interventions.

Developing this model required redefining disease control as the probability of a patient experiencing an exacerbation: high probability indicates poor control, while low probability indicates good control. Although disease control is well established for asthma [28,29,30], no universally accepted definition exists for COPD [31]. In our model, a “COPD exacerbation patient” is defined by GOLD and GesEPOC criteria: two or more moderate exacerbations or one severe exacerbation (hospitalization) in the past year. These guidelines prioritize exacerbation frequency and severity to identify high-risk patients and guide patient management [26,32], since frequent exacerbations correlate with worse prognosis and increased disease burden [33,34,35,36,37].

Given challenges in performing spirometry in primary care and the limited use of peak flow meters, lung function data and validated symptom evaluations were excluded, as they are often not recorded. Instead, we included the use of short-acting beta-agonist (SABA) inhalers as an additional metric in our control model. SABA use correlates with exacerbation risk, with higher use linked to poorer disease control and increased symptoms [38,39,40]. In patients without prior exacerbations, higher SABA use may indicate uncontrolled symptoms, potentially increasing the risk of future exacerbations.

Quantifying SABA inhaler canister use in the previous year offers a practical and sensitive measure method of symptom control for both COPD and asthma. Recent studies support this as an indirect marker of disease control and as a valuable marker for assessing current symptoms and predicting exacerbation risk [41,42]. The integration of this variable provides a comprehensive assessment of a patient’s condition, accounting for both acute events and symptom burden over time.

The criteria selected for this study—based on scientific evidence [41,42,43,44,45,46] and easily identifiable within the EHR—defined poor asthma control as either SABA use ≥3 canisters/year or ≥1 exacerbations/year [41,43,44,45]. For COPD, poor control was defined by SABA use ≥6 canisters/year, ≥2 moderate exacerbations/year, or ≥1 severe exacerbation/year (requiring hospital admission) [42,46]. Based on these criteria, these definitions were programmed into our database to automatically classify patients from Seville and Valencia as either well controlled or poorly controlled, aiming for a streamlined approach in real-world clinical settings. 

### 2.4. Statistical Analysis

Following the classification of each patient according to their disease control status (good or poor), a comprehensive statistical analysis was performed to evaluate the predictive value of each collected variable. The analysis was conducted using R software (version 4.34.2) [47], with a statistical significance level set at 95% and an alpha error of 5%.

The data structure was explored using the *inspectdf* R package, and incomplete clinical variables were excluded from the analysis (e.g., variables with more than 50% missing data and categorical variables with fewer than two categories). The development of the predictive model followed a two-phase approach: 

#### 2.4.1. Phase 1

The association between each variable and the dichotomous outcome (good/poor disease control) was evaluated using appropriate statistical tests, such as the Chi-square test and Fisher’s exact test for dichotomous variables, and the Wilcoxon signed-rank test for continuous variables. To control for Type I error, a Benjamini–Hochberg correction was applied to the combined set of *p*-values from all tests. Additionally, Spearman’s correlation matrices were used to confirm significant associations between clinical variables and disease control.

#### 2.4.2. Phase 2

Variables identified as statistically significant in Phase 1 (adjusted *p*-value < 0.05) were included in a binary logistic regression model, developed using the *glmnet* R package. A backward stepwise procedure based on the Akaike Information Criterion (AIC) *(stepAIC* function from *MASS* R package [48]) was applied to identify the most parsimonious model, which was defined as the model with the lowest AIC, indicating an optimal balance between model complexity and goodness of fit.

The model’s performance was assessed using a confusion matrix and various classification metrics, including sensitivity, specificity, positive predictive value (PPV), negative predictive value (NPV), accuracy rate, and their 95% confidence intervals. These metrics were calculated using the *confusionMatrix* function from the *caret* R package [49], with both training and validation sets (80% and 20% of the original data, respectively) to ensure robust model performance and generalizability in real-world scenarios.

In order to develop a predictive model for identifying factors associated with poor control events in patients with asthma or COPD, we divided each sample into two subsets: a training set, comprising 80% of the data, and a validation set, containing the remaining 20%. The training set was used to train the model, allowing it to learn relevant patterns and relationships within the data to predict outcomes. This prevents overfitting by ensuring the model does not become overly specific to the training data. The validation set was then employed to evaluate the model’s performance on an independent subset of data not used during training, providing an unbiased assessment of its generalizability to new data. This partitioning strategy is standard in predictive modeling to ensure a robust evaluation of model performance and applicability in real-world scenarios. 

### 2.5. Definition of Minimal Sample Size for Model Validation

To determine the minimal sample size required for identifying predictive variables of disease control with a dichotomous outcome (based on European-level data, where the incidence of the least frequent category is 28% for asthma [50,51,52] and 27.5% for COPD [27]), and considering three independent variables, the following steps were taken:

#### 2.5.1. Determination of the Minimum Number of Events in the Training Set

According to the “10 events per predictor variable” rule, the training set (80% of the original dataset) must include at least 10 events for each predictor variable to ensure stable model estimates. For three predictor variables, this necessitates a minimum of 30 events in the training set.

#### 2.5.2. Calculation of the Total Sample Size Required

To ensure both the training and validation sets contain sufficient events, the total sample size was calculated to achieve a minimum of 10 events in the validation set (20% of the dataset). Solving the equations for asthma and COPD, we find 0.2 × n × 0.28 ≥ 10 for asthma and 0.2 × n × 0.275 ≥ 10 for COPD. Resolving for both n, n ≥ 10/(0.2 × 0.28) ≈ 179 for asthma and n ≥ 10/(0.2 × 0.275) ≈ 182 for COPD.

#### 2.5.3. Verification of the Number of Events in the Training Set

It was verified that the training set (80% of the original dataset) contains more than 30 events, meeting the required threshold: 0.8 × 179 × 0.28 ≈ 40.10 for asthma and 0.8 × 182 × 0.275 ≈ 40.04 for COPD. Both calculations exceed the requirement of having at least 30 events in the training set, thereby ensuring the stability of the model during fitting.

For a predictive model with three independent variables, a minimum sample size of approximately 179 for asthma and 182 for COPD is needed to ensure proper validation, fulfilling the “10 events per predictor variable” rule for the training set and providing at least 10 events in the validation set. However, the initial sample sizes do not meet these calculated thresholds, limiting the internal validity of the predictive models. Despite this, given the novelty and significance of the topic, we have chosen to proceed with this pilot study. While the sample sizes are suboptimal for robust validation, the findings from this exploratory analysis lay a valuable foundation for future studies with larger, adequately powered samples.

## 3. Results

### 3.1. Patient Characteristics

A total of 132 asthma patients and 110 COPD patients’ EHR data were included in the exploratory data analysis for the development of a putative predictive model of poorly controlled disease. 

The baseline patient characteristics can be found in Table 1. Asthma patients were primarily located in Seville (75.8%), with the remainder from Valencia (24.2%). Conversely, COPD patients exhibited a more balanced geographic distribution between Seville (54.5%) and Valencia (45.5%). The age range for asthma patients extended from 18 to 78 years, with the most prevalent age group being 40 to 49 years. However, the age range for COPD patients was from 48 to 80 years.

Regarding disease severity, 9.1%, 1.5%, 29.5%, 38.6%, and 21.2% of asthma participants were at degrees 1, 2, 3, 4, and 5, respectively, according to the classification of GEMA 5.4 [25]. According to the 2024 GOLD classification, 16.4% of COPD patients were in group A, 40.0% in group B, and 43.6% in group E [20]. The most prevalent pharmacological treatments for asthma and COPD are listed in Table 2.

In terms of the daily frequency of inhalations, we found significant differences between the treatment recommended for asthmatic patients and for patients with COPD. Asthma patients averaged three daily inhalations, with 15.9% using one inhalation daily, 34.1% using four inhalations, and 31.8% using two inhalations. COPD patients averaged 2.5 daily inhalations, with 43.6% using 1 inhalation daily, 23.6% using 4 inhalations, and 19.1% using 2 inhalations.

Both patient groups exhibited significant data gaps in the EHR. There was a significant amount of missing information regarding height (46.2% and 58.2%), weight (75.8% and 70.0%), body mass index (BMI) (78.0% and 72.7%), obesity diagnosis (72.7% and 61.8%), and smoking status (31.8% and 27.3%) in patients with asthma and COPD, respectively.

**Figure 1:**
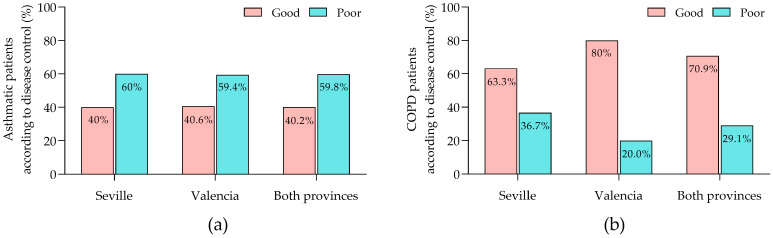
Status of disease control. (**a**) Asthmatic patients; (**b**) COPD patients. COPD, chronic obstructive pulmonary disease.

The degree of asthma control was comparable between the provinces: 40.0% of patients in Seville and 40.6% in Valencia exhibited good control, while 60.0% in Seville and 59.4% in Valencia had poor control, with no statistically significant differences between the provinces (Figure 1a). In contrast, control of COPD revealed significant provincial disparities: 63.3% of patients in Seville achieved good control compared to 80.0% in Valencia, and poor control was observed in 36.7% of patients in Seville versus 20.0% in Valencia (Figure 1b).

**Figure 2:**
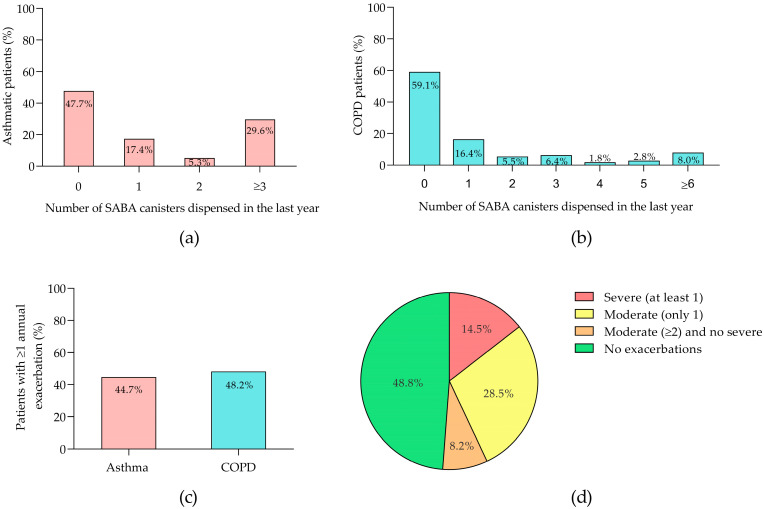
Profiles of patients with asthma and COPD. (**a**) Percentage distribution of asthmatic patients by the number of SABA canisters dispensed in the last year; (**b**) percentage distribution of COPD patients by the number of SABA canisters prescribed in the last year; (**c**) percentage of patients with asthma and COPD experiencing one or more annual exacerbations; (**d**) stratification of COPD patients by exacerbation severity. COPD, chronic obstructive pulmonary disease; SABA, short-acting β-agonist.

Analysis of SABA canister usage among asthmatic patients in the past year revealed that 47.7% used no canisters, 17.4% used one, 5.3% used two, and 29.6% used three or more (Figure 2a). Of the 70 patients prescribed SABAs, 4.3% received Terbutaline, while the rest were prescribed Salbutamol. For COPD patients, the distribution of canister usage was 59.1% for zero, 16.4% for one, 5.5% for two, 6.4% for three, 1.8% for four, 2.8% for five, and 8.0% for six or more canisters (Figure 2b). All 45 COPD patients who received SABAs were prescribed Salbutamol. Annual exacerbation rates were 44.7% for asthma and 48.2% for COPD (Figure 2c). In the COPD cohort, 18.9% of 90 exacerbations were severe. Among COPD patients, 14.5% had at least one severe exacerbation, 28.5% had two or more moderate exacerbations, 8.2% had one moderate exacerbation, and the remainder had no recorded exacerbations (Figure 2d).

Based on these results, an analysis was conducted to identify the variables most strongly associated with the degree of asthma and COPD control. 

### 3.2. Determination of Clinical Variables for Predict Poor Control of Asthma and COPD

A Spearman’s correlation analysis was performed on patient data from EHRs for both asthma and COPD to identify variables significantly associated with disease control.Results were corrected using the Bonferroni method to minimize Type I errors. For both conditions, significant variables (adjusted *p* < 0.05) included the number of SABA canisters prescribed annually, total number of SABA and SAMA canisters prescribed per year, number of annual exacerbations, number of respiratory-related consultations, number of annual prednisone courses, annual equivalent prednisone dose for lower respiratory tract problems (bronchitis or asthma attacks, in the case of asthma, or bronchitis or COPD exacerbations, in the case of COPD), number of annual antibiotic courses, and annual days of antibiotic use for lower respiratory tract problems. In addition, in the case of asthma, the type of SABA (none, Salbutamol, Terbutaline or both) was also included, and in the case of COPD, the annual number of severe exacerbations.

These correlations were validated with univariate analyses and statistical tests appropriate to each variable, followed by Bonferroni correction. Chi-square tests applied to dichotomous variables found statistical significance for the type of SABA, in the case of COPD, and the type of SAMA prescribed (none or Ipratropium Bromide; adjusted *p* < 0.05), in both pathologies, while Fisher’s exact test confirmed significance for dichotomous variables with low representation. The Wilcoxon test showed significance for continuous variables, such as the number of SABA canisters prescribed the previous year, number of SAMA canisters prescribed the previous year, total number of SABA and SAMA canisters prescribed the previous year, number of exacerbations in the previous year, number of respiratory-related consultations attended in the previous year, number of prednisone courses prescribed in the previous year for lower respiratory tract problems, total equivalent dose of prednisone in the previous year, number of antibiotic courses prescribed for lower respiratory tract problems the previous year, and number of days of antibiotic use prescribed in the previous year.

**Figure 3:**
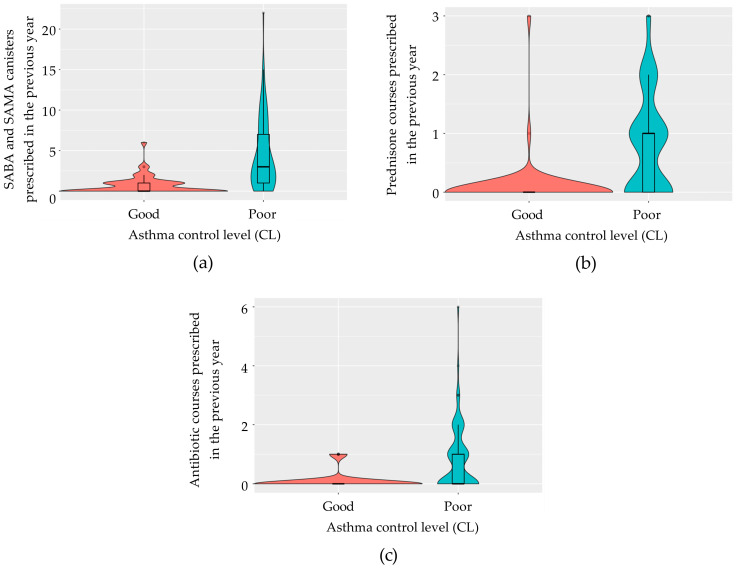
Violin plots of the predictive variables identified in asthmatic patients. (**a**) Number of SABA and SAMA canisters prescribed in the last year according to the status of disease control; (**b**) number of prednisone courses prescribed in the last year grouped by the status of disease control; (**c**) number of antibiotic courses prescribed for lower respiratory tract problems in the previous year in patients with good and poor disease control. SABA, short-acting β-agonist; SAMA, short-acting muscarinic antagonist.

**Figure 4:**
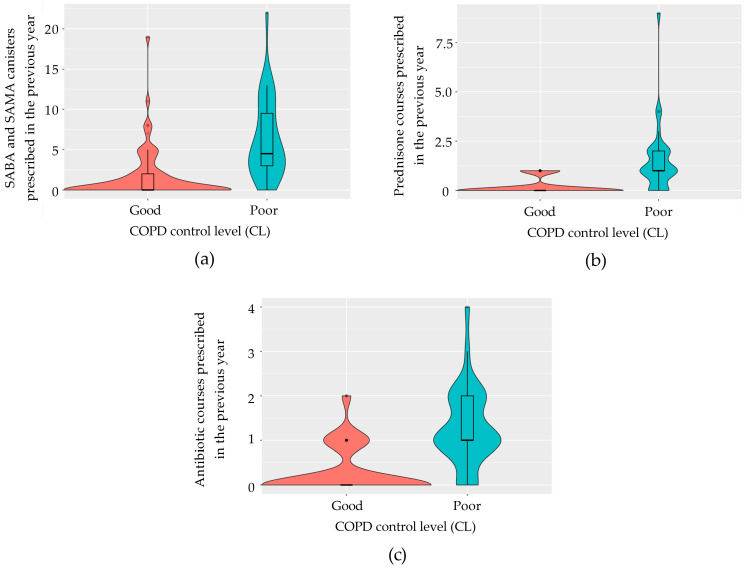
Violin plots illustrating the identified predictive variables in COPD patients according to the disease control level. (**a**) Number of SABA and SAMA canisters prescribed in the past year, comparing patients with good and poor disease control; (**b**) number of prednisone courses prescribed in the past year categorized by disease control status; (**c**) number of antibiotic courses prescribed for lower respiratory tract issues in the past year based on disease control status. COPD, chronic obstructive pulmonary disease; SABA, short-acting β-agonist; SAMA, short-acting muscarinic antagonist.

Variables used to define disease control were initially excluded from the model. Given that the calculation of variance-inflation factors (VIFs) on the model containing all pre-selected variables indicated the presence of multicollinearity, we employed a stepwise regression based on the AIC to evaluate the goodness of fit of the most simplified model, identified as the one with the lowest AIC value. For both diseases, the final models identified the total number of SABA and SAMA canisters prescribed in the previous year (Figure 3a and Figure 4a), the number of prednisone courses prescribed in the previous year (Figure 3b and Figure 4b), and the number of antibiotic courses prescribed for lower respiratory tract issues (Figure 3c and Figure 4c) as significant predictors. In asthma, the model achieved 88.5% accuracy (95% CI: 69.9–97.6), with 83.3% sensitivity, 92.9% specificity, and a kappa correlation coefficient of 76.7%. In COPD, the model achieved 86.4% accuracy (95% CI: 65.1–97.1), with 93.8% sensitivity, 66.7% specificity, and a kappa correlation coefficient of 63.7%. The predictive logistic regression coefficients for the identified variables in the model were presented in Table 3 for asthma, and in Table 4 for COPD.

However, McNemar’s test showed no significant difference between the models’ error rates and reference classifiers (asthma: *p* = 0.332; COPD: *p* = 0.302), suggesting that larger sample sizes are needed to improve the robustness and predictive accuracy. 

### 3.3. Determination of Predictive Equations for Predict Poor Control of Asthma and COPD

Predictive equations were developed for both conditions (Equation (1) for asthma and Equation (2) for COPD), where *y* represents the probability of poor control. If y < 0.50, the patient is considered to have good control; if y > 0.50, the patient is classified as having poor control, with y = 0.50 being inconclusive.

Equation (1). Predictive equation for poor control in asthma.

(1) $$y=\frac{1}{1+e^{-f\left(x\right)}}\;f\left(x\right)=-2.314+1.056\times \left[SABA+SAMA\right]+\;\;+0.022\times \left[Prednisone\_courses\right]+\;\;+1.844\times \left[Antibiotic\_courses\right].$$

*[SABA + SAMA]:* number of SABA + SAMA canisters prescribed in the last year.
*[Prednisone_courses]:* number of Prednisone courses prescribed in the last year due to bronchitis or asthma attacks.
*[Antibiotic_courses]:* number of antibiotic courses prescribed in the last year due to bronchitis or asthma attacks.

Equation (2). Predictive equation for poor control in COPD.

(2) $$y=\frac{1}{1+e^{-f\left(x\right)}}\;f\left(x\right)=-3.480+0.193\times \left[SABA+SAMA\right]+\;\;+1.814\times \left[Prednisone\_courses\right]+\;\;+1.214\times \left[Antibiotic\_courses\right].$$

[*SABA + SAMA*]: number of SABA + SAMA canisters prescribed in the past year.
[*Prednisone_courses*]: number of prednisone courses prescribed in the past year due to bronchitis or COPD exacerbations.
[*Antibiotic_courses*]: number of antibiotic courses prescribed in the past year due to bronchitis or COPD exacerbations.

## 4. Discussion

The global aging population has led to an increase in chronic diseases, with asthma and COPD being major health problems in developed countries due to their high prevalence and associated healthcare costs [53,54]. As populations continue to age, the burden of these diseases is expected to rise, necessitating effective control strategies to mitigate their impact on patients’ lives. Despite existing pharmacological and non-pharmacological strategies, many patients with asthma and COPD experience poor disease control, largely due to suboptimal medical care and the significant workload in primary care settings [12,15,55,56,57,58,59,60,61,62,63,64,65,66,67,68,69,70]. Our study aimed to develop a predictive model to estimate the preclinical probability of poor disease control using data from EHR. This tool was designed to identify patients at higher risk without requiring in-person consultations, potentially alleviating the burden on primary care.

Just as the Seles priestesses of ancient Greece predicted people’s futures by listening to the wind rustling through the leaves of oak trees (known as Zeus trees) or the tinkling of bronze vessels hanging from their branches, we have sought to interpret the signals captured in EHRs to predict the preclinical likelihood of poor disease control in patients with asthma or COPD. In analogy, we named our study Seleida, meaning “the Seles way”. This proactive approach allows for initial preclinical assessments, strategic prioritization of care, and improved clinical outcomes without overburdening primary care services [71,72]. While integrated care programs and pharmaceutical interventions have shown success in managing chronic patients by reducing exacerbations and hospitalizations [73,74,75], our EHR-based model could offer a more efficient and scalable approach.

However, this pilot study has several limitations. First, as a pilot study, the limited sample size affects the statistical power of the findings, and the results should therefore be interpreted with caution and not generalized to other populations. Second, our assumption that poor disease control in both asthma and COPD is primarily linked to exacerbations and excessive SABA use may not fully align with current guidelines, especially for COPD. Although our model was built on systematically defined, evidence-based criteria, these may differ from control definitions proposed by other authors. Additionally, the omission of symptom assessment and lung function measurements in asthma may overestimate the “good control” group while ensuring accurate classification of “poor control” patients. Third, due to the lack of a universally accepted definition of control in COPD, we adapted the asthma control concept to COPD, focusing on exacerbations and SABA use (≥6 canisters/year), as these criteria have been reliably used in similar contexts where more standardized tools like GesEPOC were not applicable [19,20,21]. However, we acknowledge that this adaptation may introduce biases that warrant further exploration in future studies. Fourth, the issue of COPD patients who have had a single moderate exacerbation in the last year remains unresolved. Can these patients be considered fully controlled? In our opinion, they cannot, but this controversial issue warrants further discussion. Moreover, the variability in EHR data recording practices across different clinical settings may influence the generalizability of our model, indicating the need for standardized data entry protocols to optimize predictive accuracy.

Our study demonstrates that predictive models using EHR data can reliably estimate poor disease control in asthma and COPD, achieving high accuracy, sensitivity, and specificity (88.5% and 86.4% accuracy; 83.3% and 93.8% sensitivity; 92.9% and 66.7% specificity for asthma and COPD, respectively). By prioritizing poorly controlled patients, healthcare resources can be managed more effectively, potentially reducing emergency visits, secondary care burdens, and associated costs. Given the limitations of current management strategies and the economic burden of uncontrolled chronic diseases [76], our model offers a promising solution to improve patient outcomes and healthcare efficiency.

Regarding the internal validity of the study, the observed proportion of poorly controlled cases in the asthma sample was 40.2%, higher than the initially estimated 28%. This higher incidence results in a sufficient number of events in both training and validation sets, satisfying the “10 events per predictor variable” rule. Thus, despite not meeting the initial sample size requirements (see Section 2.5), the observed data confirm the internal validity of the predictive model for asthma. For COPD, the observed incidence was 29.1%, slightly above the initial estimate of 27.5%. However, this increase is not enough to meet the minimum event requirement for internal validity due to sample size limitations. Nevertheless, the data from this study provide a closer approximation to real-world conditions, offering insights to inform future studies with larger, better-powered samples for further validation.

The COVID-19 pandemic underscored telemedicine’s potential as a complementary strategy in chronic disease management [77]. Integrating our predictive model with telemedicine options could further enhance individualized care for asthma and COPD patients, reducing costs and healthcare burdens while maintaining care quality [78,79]. EHR-based predictive models have been effective in other contexts, such as Alzheimer’s disease and hypertension [80,81,82,83], and could drive personalized medicine in chronic respiratory disease management [83].

The development of preclinical predictive tools enables short-, medium-, and long-term clinical strategies focused on reducing exacerbation rates by prioritizing high-risk patients. Integrating this tool into EHR systems would allow practitioners to conduct proactive assessments and optimize care.

Finally, we observed a significant data gap in EHRs for key variables like height, weight, and smoking status. This gap suggests a need for healthcare providers to ensure comprehensive data recording to support high-quality clinical practice.

This pilot study highlights the potential of EHR-based predictive models to improve the early identification of patients at risk for poor asthma and COPD control. Despite limitations in sample size and control definitions, the findings offer valuable insights for future research and more robust model development. As telemedicine and EHR-based tools become integral to healthcare, the ability to proactively manage high-risk patients could revolutionize chronic respiratory disease care, reducing healthcare burdens and enhancing outcomes. Future studies should validate these models in larger, more diverse cohorts and refine control criteria to align with evolving guidelines.

Integrating such predictive models into clinical practice could significantly advance chronic respiratory disease management. By enabling early and targeted interventions, these models can optimize patient care and resource use. With the growing emphasis on telemedicine and data-driven decisions, such tools are becoming essential for personalized and efficient care.

## 5. Conclusions

Effective predictive strategies are essential for improving patient outcomes and preventing disease progression. To the best of our knowledge, this is the first predictive model developed using real-world data from electronic medical records of asthma and COPD patients within the Spanish SNS database. The model’s high accuracy, sensitivity, and specificity make it a valuable tool for the early and precise identification of poorly controlled asthma and COPD patients in primary care, enabling proactive management without the need for in-person consultations. Following validation in broader clinical settings, primary care physicians could utilize the identified clinical variables—such as the number of SABA and SAMA canisters, prednisone courses, and antibiotic regimens in the past year—to prioritize the assessment of poorly controlled patients, conduct screenings, identify underlying causes, and adjust treatment to prevent future exacerbations.

However, the findings from this pilot study should not be yet generalizable to other populations. Future research should apply the model to a larger and more geographically diverse cohort and compare its predictions to standardized clinical assessments to validate its effectiveness. Successful validation could pave the way for integrating these predictive tools into clinical practice, potentially improving chronic disease management and optimizing healthcare resource allocation.

In conclusion, integrating real-world data-driven predictive models, such as ours, into clinical practice represents a significant advancement in chronic respiratory disease management. By leveraging electronic health record and telemedicine data, healthcare providers can make more informed and timely decisions, prioritize their interventions, and improve health outcomes for both patients and healthcare systems. As healthcare continues to embrace digital transformation, these predictive tools will become indispensable for delivering high-quality and personalized care that meets the changing needs of an aging global population.

## Figures and Tables

**Table 1:** Baseline patient characteristics.

	Asthma	COPD
**Patients (n)**	132	110
**Age (years [mean])**	50.8	68.6
**Gender**		
Male (%)	41.7	71.8
Female (%)	58.3	28.2
**Height (cm [mean])**	164	166.7
**Weight (kg [mean])**	81.1	84.6
**BMI (kg/m^2^ [mean])**	30.8	30.9
**BMI ≥ 30 kg/m^2^ (%)**	13.6	26.4
**Smoking habits**		
No smoker (%)	40.9	0.0
Current smoker (%)	17.4	49.1
Former smoker (%)	9.9	23.6
Unknown (%)	31.8	27.3
**Allergies**		
Drugs (%)	20.5	14.5
Environmental (%)	57.6	4.5
Food (%)	4.5	1.8

“Unknown” corresponds to the absence of data on electronic healthcare records (EHRs). BMI, body mass index; COPD, chronic obstructive pulmonary disease.

**Table 2:** Patient treatment profile.

	Asthma	COPD
**Treatment regimens**		
No treatment (%)	1.5	3.6
SABAs, SAMAs or both (%)	7.6	3.6
LABAs (%)	0.0	0.9
LAMAs (%)	0.0	14.5
LABAs + LAMAs (%)	0.0	30.0
**ICs**		
Low-dose (%)	0.8	
Medium-dose (%)	0.8	0.0
High-dose (%)	0.8	
**LABAs + ICs**		
Low-dose ICs (%)	25.0	
Medium-dose ICs (%)	25.0	15.5
High-dose ICs (%)	8.3	
**LAMAs + LABAs + ICs**		
Low-dose ICs (%)	0.0	
Medium-dose ICs (%)	0.8	30.0
High-dose ICs (%)	3.0	
**LTRAs + LABAs + ICs**		
Low-dose ICs (%)	3.8	
Medium-dose ICs (%)	9.1	0.0
High-dose ICs (%)	6.1	
**Daily inhalations (number [mean])**	3.0	2.5
**Patients according to the number of daily inhalations**		
1 (%)	15.9	43.6
2 (%)	31.8	19.1
4 (%)	34.1	23.6
Other (%)	<6	<5

COPD, chronic obstructive pulmonary disease; ICs, inhaled corticosteroids; LABAs, long-acting beta-agonists; LAMAs, long-acting muscarinic antagonist; LTRAs, leukotriene receptor antagonists; SABAs, short-acting β-agonists; SAMAs, short-acting muscarinic antagonists.

**Table 3:** Coefficients of selected asthma variables.

	Estimate	Std. Error	z Value	Pr (>|z|)
(Intercept)	−2.313598	0.560061	−4.131	0.000036 ***
SABA + SAMA canisters	1.055785	0.292446	3.610	0.000306 ***
Prednisone courses	0.021774	0.008762	2.485	0.012949 *
Antibiotic courses	1.843722	0.767671	2.402	0.016319 *

* *p* < 0.05; *** *p* < 0.001. SABA, short-acting β-agonist; SAMA, short-acting muscarinic antagonist.

**Table 4:** Coefficients of selected chronic obstructive pulmonary disease (COPD) variables.

	Estimate	Std. Error	z Value	Pr (>|z|)
(Intercept)	−3.47968	0.71398	−4.874	0.000001 ***
SABA + SAMA canisters	0.19257	0.09767	1.972	0.04865 *
Prednisone courses	1.81380	0.61276	2.960	0.00308 **
Antibiotic courses	1.21442	0.52236	2.325	0.02008 *

* *p* < 0.05; ** *p* < 0.01; *** *p* < 0.001. SABA, short-acting β-agonist; SAMA, short-acting muscarinic antagonist.

## Data Availability

The original contributions presented in the study are included in the article; further inquiries can be directed to the corresponding authors.
